# Design of a Miniaturized Rectangular Multiturn Loop Antenna for Shielding Effectiveness Measurement

**DOI:** 10.3390/s20113178

**Published:** 2020-06-03

**Authors:** Sangwoon Youn, Tae Heung Lim, Eunjung Kang, Dae Heon Lee, Ki Baek Kim, Hosung Choo

**Affiliations:** 1 School of Electronic and Electrical Engineering, Hongik University, Seoul 04066, Korea; tirano88@naver.com (S.Y.); qpzm_0105@naver.com (T.H.L.); lemon_ya@naver.com (E.K.); 2 Affiliated Institute of Electronics and Telecommunications Research Institute, Daejeon 30147, Korea; leedh@nsr.re.kr (D.H.L.); kbkim11@nsr.re.kr (K.B.K.)

**Keywords:** VLF antenna miniaturization, ferrite loop antenna, multiturn loop antenna, AF enhancement, SE measurement

## Abstract

This paper proposes a novel miniaturized rectangular loop antenna sensor consisting of a multiturn wire and a cuboid ferrite core. The lateral surface of the ferrite core is tightly wound by the multiturn wire. To verify its feasibility, the antenna sensor is fabricated, and the antenna factor (AF) levels are measured using the three-antenna method from the very low frequency (VLF) to the high-frequency (HF) bands. The measured AF levels are 31.8 dB (with a covering plastic case) and 33.1 dB (without a covering plastic case) at 30 kHz. In addition, the proposed antenna is employed in the shielding effectiveness measurement of a small commercial cabinet to observe its suitability for shielding effectiveness (SE) measurement of small shielding enclosures. The SE values averaged over the frequency range from 10 kHz to 3 MHz are 4.1 dB and 12 dB in the horizontal and vertical polarizations, respectively.

## 1. Introduction

In recent electronic warfare, shielding facilities have become essential to protect important electrical devices from electromagnetic pulse (EMP) attacks [1,2,3]. For low-cost maintenance, small shielding enclosures that can only shield electrical devices have been gradually demanded to protect these devices more efficiently [4,5,6,7]. Shielding effectiveness (SE) for such small shielding enclosures also needs to be measured at various frequency ranges to determine the EMP strength that the enclosures can endure. In conventional SE measurements based on IEEE standard 299.1, loop antennas are generally used to measure SE from the very-low-frequency (VLF) band to the high-frequency (HF) band in a large shielding room [8,9,10,11,12]. However, SE measurements of small shielding enclosures are significantly problematic using conventional SE measurement systems due to the large size of the loop antenna [13]. For example, the small shielding enclosure usually contains one side of the enclosure that is less than 700 mm according to the IEEE standard 299. 1. However, it is difficult to accurately measure the SE levels at edges or sides of the small shielding enclosure, because the physical size of the conventional loop antenna sensor, i.e., AH-systems [14], has a large diameter of 300 mm. To overcome this issue, research on miniaturization techniques for loop antennas has been extensively conducted by employing various loop designs [15,16,17,18], printed loops with ground planes [19], and adding lumped elements [20,21,22]. Although these approaches can miniaturize the physical size of loop antennas, such techniques usually require sophisticated and expansive fabrication processes. In addition, these loop antennas often have high antenna factors (AFs) in the VLF band, which can degrade the accuracy of the SE measurement. Thus, to improve the accuracy of the SE measurement, various antenna sensor structures have been introduced [23,24], such as shielded loop antennas [25], two-port loop antennas [26], loop antennas with parasitic elements [27], and Moebius loop antennas [28]. These previous studies have been successful in improving measurement accuracy with antenna sensors operating in a narrow frequency band. However, the SE measurement using such narrow band antenna sensors requires a lot of manpower and time, because the antenna sensors need to be replaced depending on the operating frequency bands. In addition, there is still a need for in-depth research on the miniaturization of the loop antenna with broadband characteristics in the VLF band, which can maintain low AF levels.

In this paper, we propose a novel design structure of a compact rectangular loop antenna sensor composed of a multiturn wire and a cuboid ferrite core. The proposed antenna has a cuboid-shaped ferrite core wound multiple times by a thick wire to concentrate the magnetic flux density, which can reduce the AF level in the VLF band. Then, the antenna characteristics such as the impedance and the AF in terms of the frequency are estimated using the FEKO EM simulator [29]. The theoretical AF is also derived by the multiturn loop formula and compared to the simulation results. To verify the feasibility, the proposed antenna sensor is fabricated to measure its AF characteristics using the three-antenna method from the VLF to HF bands. The variations in the AFs according to the frequency are observed depending on the important antenna geometry parameters. The antenna sensor is finally utilized to carry out the SE measurement of the commercial small metallic cabinet, and the results demonstrate that it is suitable for measuring the SE of small shielding enclosures from VLF to HF bands.

## 2. Proposed Antenna and Theory

### 2.1. Theoretical Background

**Figure 1:**
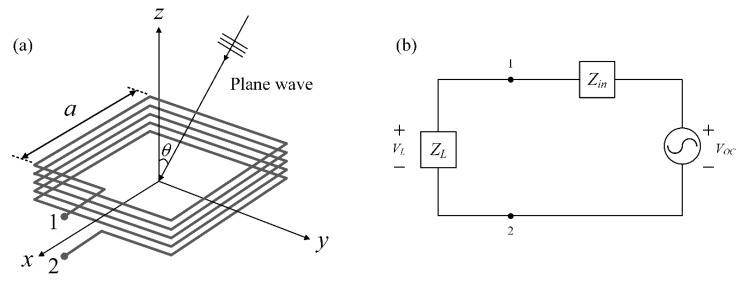
Conceptual figure of the rectangular multiturn loop antenna: (**a**) planewave toward the rectangular loop antenna; (**b**) thevenin equivalent circuit model of the loop antenna.

Figure 1a illustrates a conceptual geometry of the rectangular multiturn loop antenna in a receiving mode with a side length of *a* and *N* turns. When an electromagnetic planewave propagates toward the loop antenna at the incident angle of *θ*, an open-circuit voltage is then induced across nodes 1 and 2. To easily understand this concept, the receiving loop antenna can be described by a Thevenin equivalent circuit as shown in Figure 1b, and the circuit components are composed of an input impedance of *Z_in_*, a load impedance *Z_L_* of 50 Ω, and an open-circuit voltage of *V_oc_*. As written in Equation (1), the open-circuit voltage *V_oc_* can be expressed by an area of the physical loop antenna size and the amplitude of the magnetic field intensity [30,31]. The load voltage *V_L_* can be calculated by the voltage ratio of the input and load impedances as expressed in Equation (2). Then, the AF of the loop antenna is derived by the ratio between the magnetic field intensity and the loaded voltage as noted in Equation (3) [32].

(1) $$V_{oc}=j\omega a^{2}\mu_{0}HN\sin\theta ,$$

(2) $$V_{L}=V_{oc}\frac{Z_{L}}{Z_{in}+Z_{L}},$$

(3) $$\mathrm{AF}=20\log_{10}(H/V_{L}).$$

**Figure 2:**
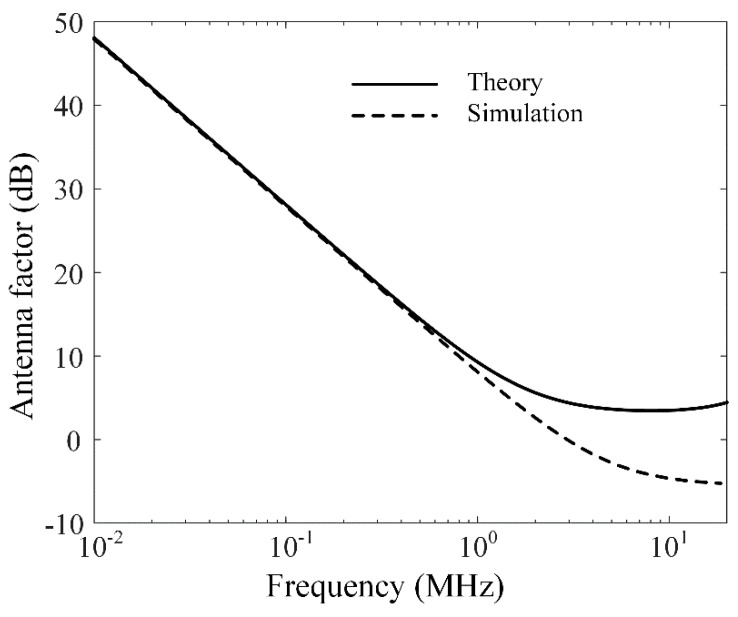
Antenna factor (AF) comparisons of the theoretical result and simulation.

To reconfirm the theoretical result, we model the multiturn loop antenna using the FEKO EM simulator based on the method of moments (MoM) with piecewise tetrahedron meshes of 1/1000 wavelength at 20 MHz. The antenna has a rectangular loop structure with a side length of *a* and *N* = 5. The simulation model was analyzed with piecewise tetrahedron meshes of 1/1000 wavelength for 20 MHz. We also applied double precision option and low-frequency stabilization to obtain the suitable numerical simulation results. Figure 2 presents AF comparisons of the theoretical result and simulation according to frequency. The theoretical result is derived by Equations (1)–(3) using the input impedance of the loop antenna, which is indicated by the solid line. The dashed line shows the simulation result obtained using the magnetic field intensity and loaded voltage under the same conditions as in theory. AFs by the theoretical and simulation results show a similar trend in most of frequencies, with values of 38.8 dB and 38.6 dB at 30 kHz, respectively. However, the AF levels for both results have a slight difference in the high-frequency band because the loop antenna model in the equation makes it difficult to consider the detailed antenna parameters.

### 2.2. Proposed Antenna Structure and Simulation

**Figure 3:**
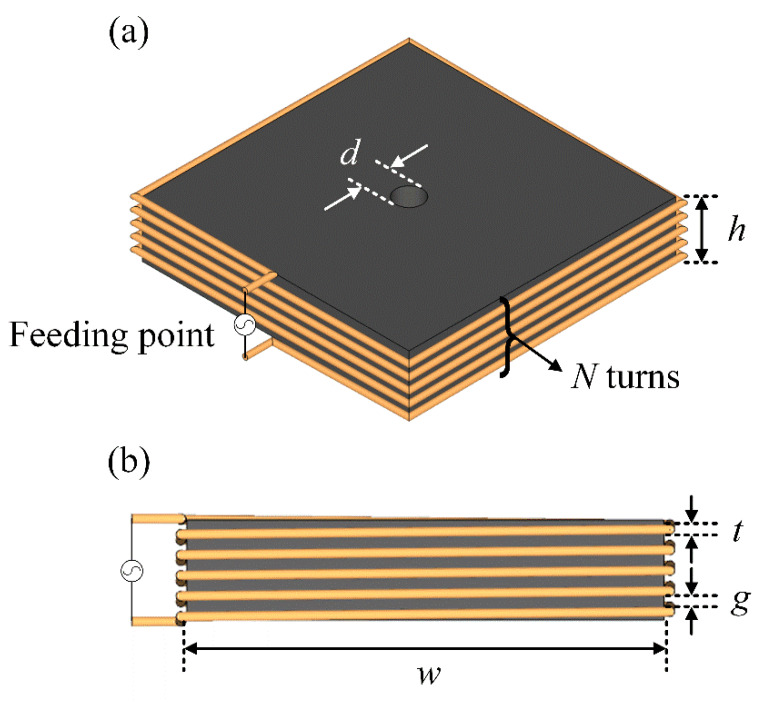
Geometry of the proposed antenna: (**a**) isometric view; (**b**) side view.

**Figure 4:**
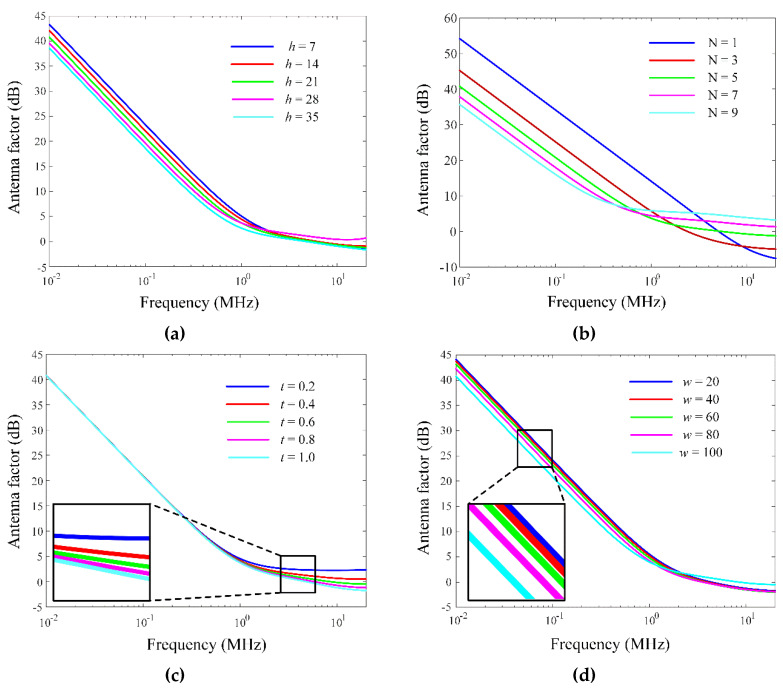
AF variation according to design parameters: (**a**) AF in accordance with the variation of *h*, (**b**) AF in accordance with the variation of *N*, (**c**) AF in accordance with the variation of *t*, and (**d**) AF in accordance with the variation of *w*.

Figure 3a,b show the geometry of the rectangular multiturn loop antenna sensor. The proposed antenna consists of a rectangular multiturn loop and a cuboid-shaped ferrite core (*μ_r_* = 210). The cuboid-shaped ferrite core, having a thickness of *h* with a width of *w*, is wound by a thick wire with a diameter *t*, which can miniaturize the antenna’s physical structure size with a low AF in the VLF band. In addition, the *N* turns of the rectangular loop wire helps to concentrate a strong magnetic flux density in the center of the antenna. The detailed design parameters are listed in Table 1. Figure 4a–d show the AF results in accordance with the variations of the important geometry parameters (*h*, *N*, *t*, and *w*). As can be seen in Figure 4a, the AF levels in the entire frequency range decrease due to the ferrite property that focuses the magnetic flux density in the center of the antenna when the ferrite thickness *h* increases. Figure 4b presents that the AF levels dramatically decrease at low frequencies, but AF tends to increase at high frequencies, when the number of the wire turns increases. Figure 4c illustrates the AF levels in accordance with the variation of the wire diameter *t*. The AF levels in the HF bands are reduced as *t* increases, because the low input impedance characteristic at the high frequency can induce the high load voltage as indicated in Figure 1b. The low AF levels are obtained as the ferrite core width *w* increases, as shown in Figure 4d. Note that the magnetic flux passing through the center of the ferrite core becomes dense and can lead to the high induced open-circuit voltage. The resulting parametric study demonstrates that the proposed antenna sensor can be miniaturized by the ferrite core with the multiturn thick wire.

## 3. Antenna Fabrication and Measured Results

**Figure 5:**
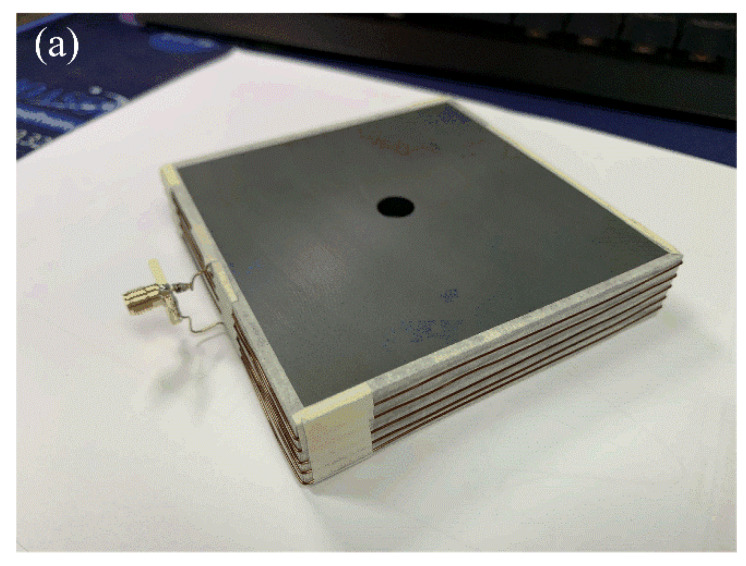

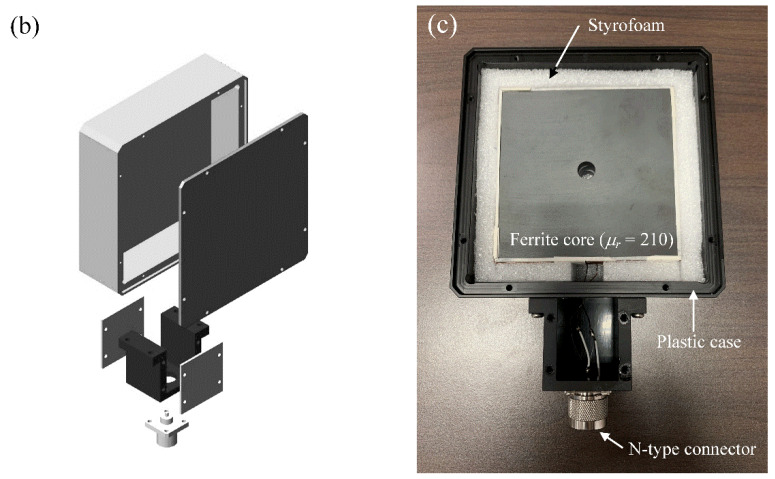
Photographs of the fabricated and assembled antenna: (**a**) isometric view of the proposed antenna, (**b**) casing parts, and (**c**) assembled antenna.

**Figure 6:**
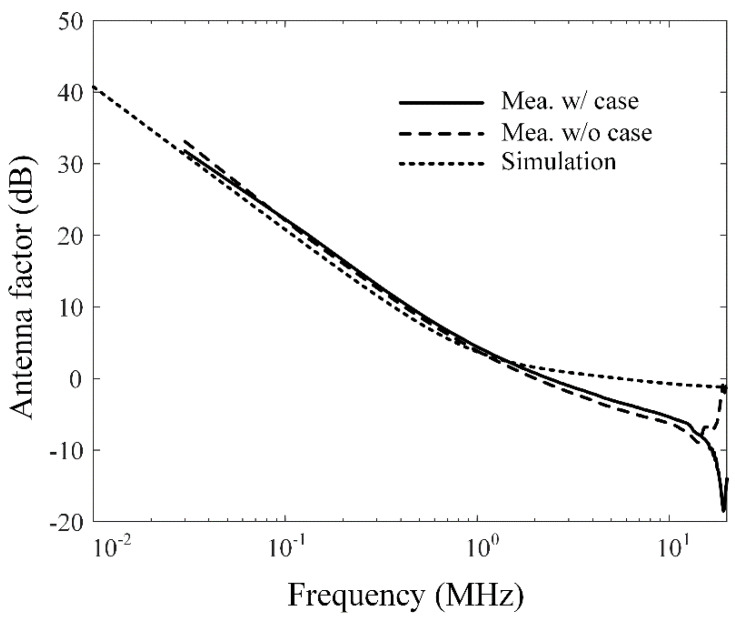
AF comparisons of the simulation and measurements.

**Figure 7:**
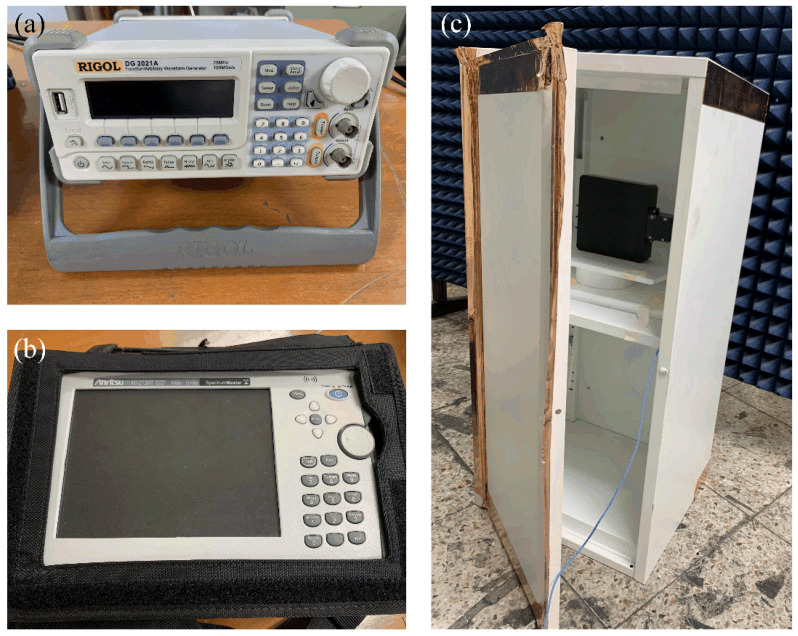
SE measurement setup: (**a**) function generator; (**b**) spectrum analyzer; and (**c**) loop antenna within the commercial metallic cabinet.

**Figure 8:**
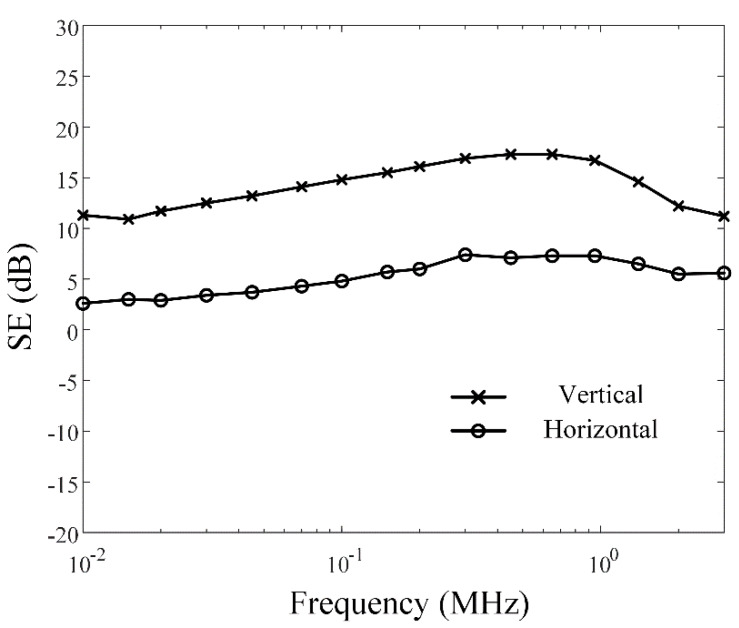
Measured SE in horizontal and vertical polarizations.

Figure 5a–c present photographs of the fabricated antenna sensor, a covering plastic case, and an assembled structure. The multiturn loop antenna has a simple structure, which is composed of the copper wire coated by polyethylene and the ferrite core (SD 100 × 100 × 7, Samwha). The lateral surfaces of the cuboid-shaped ferrite are wound by the wire with five turns, which are firmly wrapped by Styrofoam to protect the antenna from external shocks. Then, this fabricated antenna is inserted in the covering plastic case being assembled with nuts to improve durability, and the ends of the multiturn loop are directly connected to the N-type connector as a feeder. The covering case makes it easy to handle and enables practical SE measurements. To examine AF characteristics, the fabricated antenna is measured by applying the three-antenna method [33]. In this method, S-parameters are obtained by utilizing two reference antennas and one test antenna. For two reference antennas, we fabricate two single-turn circular wire loop antenna having a loop diameter of 30 cm with a SMA feeder. Figure 6 represents the measured and simulated AF levels of the proposed rectangular multiturn antenna. The measured AF values with and without the covering plastic case are 31.8 dB and 33.1 dB and agree with the simulation of 30.9 dB at 30 kHz. The slight difference of the AF levels between the simulations and measurements in high frequency occurs due to the fabrication error, insufficient material parameters, and simplified feeding structure. We also compare the antenna performances, such as operating frequency bands, antenna geometry dimensions, and AF levels at 30 kHz, between the proposed antenna sensor and the reference loop antennas. The detailed values are listed in Table 2. The results confirm that the proposed sensor can maintain low AF values from the VLF band to the HF band despite the small physical antenna size, which is downsized to 33% of the conventional loop antenna. We additionally carried out the SE measurement of a commercial metallic cabinet using the proposed antenna in order to confirm the feasibility. In IEEE standard 299.1, SE is defined as an indicator of the shielding performance for an enclosing structure, which can be obtained by comparing the field intensities with and without the shielding structure from an external electromagnetic source. Figure 7a–c show the SE measurement setup with measurement equipment such as a function generator, a spectrum analyzer, and a power amplifier. We use the function generator that is connected to the proposed antenna and the power amplifier with a gain of 18 dB for the transmitting part. For the receiving part, the identical antenna is connected to the spectrum analyzer to measure the field strength. Figure 8 shows the result of the SE measurement of the metallic cabinet by the vertical and horizontal polarization, respectively. The cabinet has average SE values of 12 dB for a vertical polarization and 4.1 dB for a horizontal polarization.

## 4. Conclusions

In this paper, we proposed a novel design structure of a compact rectangular loop antenna composed of a multiturn wire and a cuboid ferrite core. The antenna sensor had a cuboid-shaped ferrite core wound by a thick wire in order to concentrate the magnetic flux density in the center of the antenna, which could reduce the AF levels in the VLF band. The measured AF levels of the proposed antenna were 31.8 dB (with a covering plastic case) and 33.1 dB (without a covering plastic case) at 30 kHz, which agreed well with the simulation of 30.9 dB. The SE measurement results of the commercial cabinet over the frequency range from 10 kHz to 3 MHz were 4.1 dB and 12 dB in the horizontal and vertical polarizations, respectively.

## Figures and Tables

**Table 1:** Design parameters of the proposed antenna.

Parameters	Values
*t*	1 mm
*g*	4 mm
*h*	21 mm
*w*	100 mm
*d*	10 mm
*N*	5 turns

**Table 2:** Antenna performance comparison.

Antenna	Frequency	Dimension (mm)	AF (@30kHz)
[10]	9 kHz–10 MHz	60 × 60 × 6	-
[30]	1 MHz–30 MHz	152 × 152 × 3.7	-
A.H. system (SAS-563P) [14]	10 kHz–30 MHz	300 × 300	28 dB
ETS-lindgren (6512) [34]	9 kHz–30 MHz	600 × 600 × 38	27 dB
TDK (LP-0930P) [35]	9 kHz–30 MHz	600 × 600	21 dB
Proposed Ant.	10 kHz–30 MHz	100 × 100 × 21	31.8 dB
